# Functional characterization of the PI3K/AKT/MTOR signaling pathway for targeted therapy in B-precursor acute lymphoblastic leukemia

**DOI:** 10.1038/s41417-022-00491-0

**Published:** 2022-07-06

**Authors:** Patricia K. Grüninger, Franziska Uhl, Heike Herzog, Gaia Gentile, Marta Andrade-Martinez, Tobias Schmidt, Kyuho Han, David W. Morgens, Michael C. Bassik, Michael L. Cleary, Oliver Gorka, Robert Zeiser, Olaf Groß, Jesús Duque-Afonso

**Affiliations:** 1 Department of Hematology/Oncology/Stem Cell Transplantation, University of Freiburg Medical Center, Faculty of Medicine, University of Freiburg, Freiburg, Germany; 2 Department of Genetics, Stanford University School of Medicine, Stanford, CA USA; 3 Department of Pathology, Stanford University School of Medicine, Stanford, CA USA; 4 Institute of Neuropathology, University of Freiburg Medical Center, Faculty of Medicine, University of Freiburg, Freiburg, Germany; 5 Signalling Research Centres BIOSS and CIBSS, University of Freiburg, Freiburg, Germany; 6 Center for Basics in NeuroModulation (NeuroModulBasics), University of Freiburg Medical Center, Faculty of Medicine, University of Freiburg, Freiburg, Germany

**Keywords:** Cell biology, Targeted therapies

## Abstract

B-cell precursor acute lymphoblastic leukemias (B-ALL) are characterized by the activation of signaling pathways, which are involved in survival and proliferation of leukemia cells. Using an unbiased shRNA library screen enriched for targeting signaling pathways, we identified MTOR as the key gene on which human B-ALL E2A-PBX1^+^ RCH-ACV cells are dependent. Using genetic and pharmacologic approaches, we investigated whether B-ALL cells depend on MTOR upstream signaling pathways including PI3K/AKT and the complexes MTORC1 or MTORC2 for proliferation and survival in vitro and in vivo. Notably, the combined inhibition of MTOR and AKT shows a synergistic effect on decreased cell proliferation in B-ALL with different karyotypes. Hence, B-ALL cells were more dependent on MTORC2 rather than MTORC1 complex in genetic assays. Using cell metabolomics, we identified changes in mitochondrial fuel oxidation after shRNA-mediated knockdown or pharmacological inhibition of MTOR. Dependence of the cells on fatty acid metabolism for their energy production was increased upon inhibition of MTOR and associated upstream signaling pathways, disclosing a possible target for a combination therapy. In conclusion, B-ALL are dependent on the PI3K/AKT/MTOR signaling pathway and the combination of specific small molecules targeting this pathway appears to be promising for the treatment of B-ALL patients.

## Introduction

Acute lymphoblastic leukemia (ALL) is the most common malignancy in children, representing 22% of cancers below the age of 18 years [1]. Precursor B-ALL (B-ALL) is the most frequent subtype in pediatric ALL and is characterized by a block in differentiation of early lymphoid precursor cells. Several ALL subtypes have been described based on their karyotype, cell type, immunophenotype and gene expression profile. The pre-BCR subtype is characterized by the expression of a pre-B cell receptor (pre-BCR) [2]. Interestingly, half of the cases are associated with the chromosomal translation t(1;19), coding for the chimeric fusion protein E2A-PBX1 [3], which is present in about 5% of pediatric and adult ALL [4]. Recent studies have shown that E2A-PBX1^+^ B-ALL cells are dependent on signaling pathways associated with the pre-BCR receptor [2, 5–8]. The dependence on these signaling pathways has been confirmed in other B-ALL subtypes [9].

The peak of incidence of ALL is at the age of 1–4 years, it then decreases before gradually increasing again around the age of 50 [10]. Although the 5-year survival rate in childhood ALL is currently about 90% using conventional chemotherapies, 7% of the patients suffer a secondary neoplasm. Hence, long-term studies show an elevated risk of mortality due to recurrence or progression, second primary cancer, circulatory or pulmonary disease [1, 11]. Furthermore, development of resistance to chemotherapy has been observed frequently. In adults, comorbidities can limit the therapy and the 5-year survival rate is about 40%, mostly due to relapses [12]. Therefore, less toxic but more effective therapeutic strategies are urgently needed in this patient population.

The excellent improvements in the prognosis of ALL patients during the last decades are mostly due to the optimizations of dose and schedule of intensified therapy regimes [13]. Powerful new techniques (proteomics, metabolomics, and next-generation sequencing) have supported the development of new therapies in ALL focusing on targeting specific kinases and signaling pathways, which should further improve the prognosis of individual patients and reduce toxicities compared to conventional chemotherapy [14, 15].

In this study we identified MTOR in an unbiased shRNA library screen as the top gene on which B-ALL cells, driven by the chimeric fusion protein E2A-PBX1, are dependent for proliferation. We examined the effect of several small molecule inhibitors inhibiting the PI3K/AKT/MTOR signaling pathway in B-ALL cell lines. The MTOR-inhibitor torin-1 synergized with the AKT-inhibitor capivasertib (AZD5363) in the inhibition of the proliferation of B-ALL cells. Furthermore, we identified changes in mitochondrial fuel oxidation of B-ALL after shRNA-mediated knockdown or pharmacological inhibition of MTOR, which could be exploited therapeutically.

## Results

### Identification of key genes and pathways in E2A-PBX1^+^ leukemia cells

**Fig. 1:**
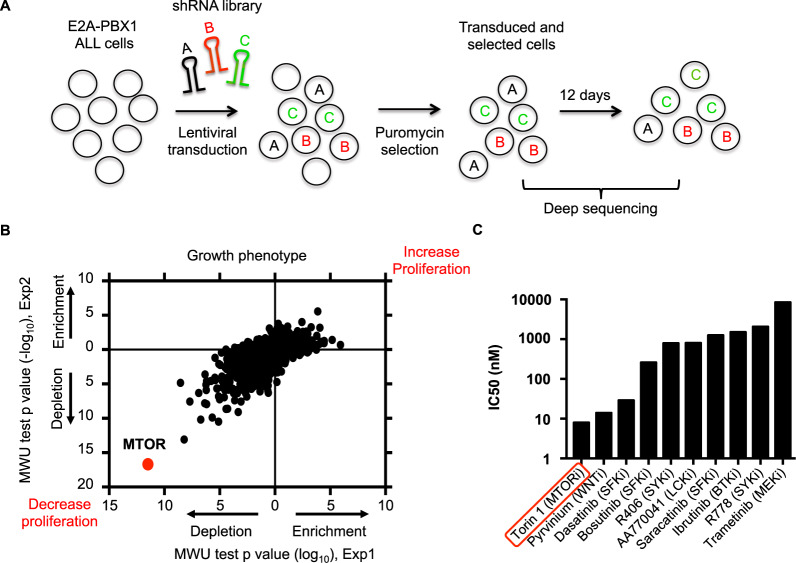
Identification of key genes using a shRNA library screen approach. **A** Schematic representation of the shRNA library screen. RCH-ACV leukemia cells (E2A-PBX1^+^/pre-BCR^+^) were transduced with shRNA sublibraries. After puromycin selection, the frequency of individual shRNAs was quantified at day 0 and at day 12 by deep sequencing. The experiment was performed in duplicate. **B** Dot plot shows Mann–Whitney *U* test *p* values for enrichment (increase proliferation) and depletion (decrease proliferation) of targeted genes by shRNA knockdown and analyzed by deep-sequencing. Each dot represents a gene. **C** Small drug screen. RCH-ACV cells were treated with several small molecule inhibitors and viable cells were enumerated after four days. Graph shows half inhibitory growth concentration (IC50). At least three independent experiments were performed per treatment.

We employed a functional genomics approach based on ultra-complex short hairpin RNA (shRNA) library screening to identify novel genes and pathways, on which human E2A-PBX1^+^ ALL cells are strongly dependent [16]. This technical approach was used to systematically create specific depletions of targeted transcripts in human E2A-PBX1^+^ ALL RCH-ACV cell line, which were then evaluated for effects on sustained proliferation (enhanced or suppressed) over time (Fig. 1A). Bioinformatic analysis identified *MTOR* as the top gene, on which E2A-PBX1^+^ cells are dependent for proliferation (Fig. 1B, Supplementary Table S1–S2). Besides *MTOR*, we identified among the top 5 genes decreasing cell proliferation *SMC3, PSMD1, RAD21 and RPA3*. The top 5 genes increasing cell proliferation were *PTEN, BMPR2, CALM2, PTPRG* and *PIK3C3* (Supplementary Table S1). Several PI3K/AKT/MTOR pathway genes were identified to affect cell proliferation in the shRNA screen (e.g. *PIK3CD, AKT2, AKT1, RPS6*) (Supplementary Table S3) confirming our previous data about PI3K/AKT/MTOR activation in mouse E2A-PBX1^+^ leukemia cells [6]. Identified kinases susceptible to pharmacologic inhibition were validated using biochemical assays. Remarkably, the novel specific enzymatic MTOR inhibitor torin-1 was the most potent compound in a small-scale drug screen using several small molecule inhibitors in E2A-PBX1^+^/preBCR^+^ ALL cells, validating the shRNA screen data using an independent pharmacologic approach (Fig. 1C).

### RICTOR, but not RPTOR, is essential for the proliferation of E2A-PBX1^+^ ALL cells

**Fig. 2:**
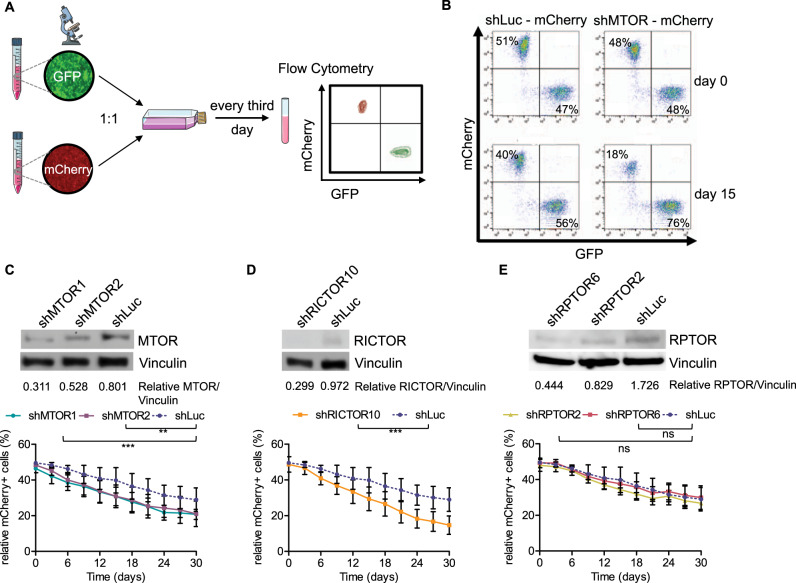
Depletion of MTOR or RICTOR decreases proliferation of human ALL cells. **A** Scheme of proliferation competition assay. Parts of the image are taken from https://smart.servier.com. **B** Dot plot proportion of mCherry^+^ and GFP^+^ cells in flow cytometry at day 0 and after 15 days of culture of a representative experiment, in which RCH-ACV cells were transduced with control shRNA (shLuc, Luciferase) or shRNA for MTOR (shMTOR) with a mCherry as fluorescence marker. RCH-ACV cells transduced with control shRNA (shLuc) with a GFP marker were used as control cells in the competition assay and mixed 1:1 at day 0. **C**–**E** Western blot analysis (representative of three independent experiments) at the top of the panel show protein levels following knockdown by different shRNA constructs in RCH-ACV cells. Vinculin was used as loading control. Densitometry values were calculated using ImageJ software. Diagrams at the bottom show percentage of mCherry^+^ cells transduced with shRNAs for luciferase (control) or different shRNA constructs of (**C**) MTOR, (**D**) RICTOR or (**E**) RPTOR for 30 days. Data represent the mean ± SEM of three independent experiments. Statistical analysis performed using non-linear regression analysis and curves were compared with the sum-of-squares F test. ns not significant, **p < 0.01, ***p < 0.001.

To validate the genetic screen, we employed single shRNA sequences, which specifically depleted *MTOR* in RCH-ACV cells and performed proliferation competition assays (Fig. 2A, B). Hence, to investigate which upstream complexes (MTORC1 or MTORC2) are involved in cell survival and proliferation, we depleted rapamycin-independent companion of MTOR (*RICTOR)* and regulatory-associated protein of MTOR (*RPTOR)* using single shRNAs in E2A-PBX1^+^ B-ALL cells (Supplementary Table S4).

Depletion of *MTOR* showed a relevant effect on cell proliferation (Fig. 2C, Supplementary Fig. S1), validating the shRNA library screen assay. Notably, *RICTOR*-depleted B-ALL cells had a proliferative disadvantage compared to control B-ALL cells. The percentage of cells with a depletion of *RICTOR* was decreased by 34% compared with control transduced cells over 30 days in culture (Fig. 2D). In contrast, knockdown of *RPTOR* had the same effect on cell proliferation as the control vector (Fig. 2E). These data suggest that RCH-ACV cells are strongly dependent on MTOR and RICTOR (MTORC2 complex) but not on RPTOR (MTORC1 complex) for proliferation and survival.

### Torin-1 is a potent inhibitor of proliferation in human pre-B ALL cells

**Fig. 3:**
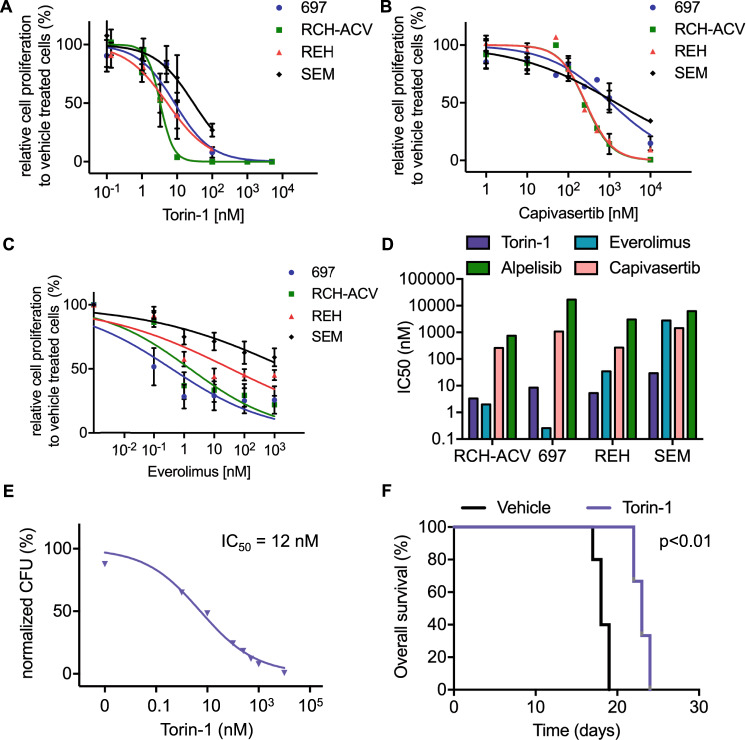
Human ALL cell lines are susceptible to pharmacological inhibition of the PI3K/AKT/MTOR signaling pathway. Titration curves for human leukemia cells cultured with increasing concentrations of (**A**) torin-1, (**B**) capivasertib and (**C**) everolimus. Viable cells were counted with trypan blue exclusion assay after four days. Data represent mean ± SEM of three independent experiments. **D** Graph shows means of half inhibitory growth concentration (IC50) of three independent experiments. **E** Titration curves of torin-1 for colony forming assay using murine E2A-PBX1^+^/PreBCR^+^ leukemia cells. Data represent mean ± SEM of three independent experiments. CFU, colony forming units. **F** Kaplan–meier curve represent disease-free survival of recipient mice transplanted with murine E2A-PBX1^+^/PreBCR^+^ leukemia cells after sublethal irradiation and treatment with vehicle or torin-1 (20 mg/kg b.w./d) starting at day 8 after transplantation. Each cohort contains 5 mice. Statistical analysis was performed by log-rank test.

We tested several PI3K, AKT and MTOR inhibitors, most of which are FDA-approved or in clinical trials for the treatment of hematological malignancies or solid tumors, in the human B-ALL cell lines RCH-ACV, 697, REH and SEM. All tested inhibitors decreased cell viability in a dose-dependent manner (Fig. 3A–C, Supplementary Fig. S2).

The MTOR-enzymatic inhibitor torin-1 efficiently suppressed cell proliferation in all tested cell lines, with an IC_50_ between 3 and 30 nM (Fig. 3A, D). Cell lines RCH-ACV and REH showed nearly the same sensitivity to AKT inhibitor capivasertib, with an IC_50_ of 260 nM (Fig. 3B, D).

The PI3K inhibitors alpelisib and idelalisib showed an inhibitory effect on cell proliferation only at high doses in B-ALL cell lines. For ipatasertib, an AKT inhibitor, the IC50 was above 1000 nM in three out of four tested B-ALL cell lines (Supplementary Fig. S2).

The E2A-PBX1^+^ cell lines RCH-ACV and 697 were more sensitive to the allosteric MTOR inhibitor everolimus, than E2A-PBX1^−^ cell lines (Fig. 3C). However, we did not observe a consistent difference in sensitivity to AKT- and PI3K-inhibitors between E2A-PBX1^+^ and E2A-PBX1^−^ cell lines (Supplementary Fig. S3).

Using mouse E2A-PBX1+/preBCR+ leukemia cells [6], we tested several inhibitors targeting the PI3K/AKT/MTOR pathway. Mouse E2A-PBX1+/preBCR+ leukemia cells were sensitive to the torin-1 treatment with an IC50 of 12 nM (Fig. 3E). Hence, the mouse leukemia cells were moderate sensitive to everolimus with an IC50 of 497 nM. In contrast to human ALL cells, the mouse E2A-PBX1+/preBCR+ leukemias showed resistence to the inhibitory effects of capivasertib with an IC50 of about 10 µM. (Supplementary Fig. S4A, B).

The preclinical efficacy of torin-1 was tested in vivo after transplantation of murine E2A-PBX1^+^/preBCR^+^ leukemia cells [6]. As expected, in vivo treatment with torin-1 prolonged disease-free survival compared to vehicle-treated mice (Fig. 3F, median disease-free survival for vehicle 18 days; for torin-1 24 days, *p* < 0.01). Hence, recipient mice treated with torin-1 showed less leukocytosis, thrombocytopenia and anemia as well as less pronounced hepatosplenomegaly and lymphadenopathy compared to vehicle-treated mice (Supplementary Fig. S5A–F). The infiltration of leukemia cells, quantified by GFP-positive cells in flow cytometry, was also reduced in torin-1- compared to vehicle-treated-mice in several analyzed tissues and organs, including the central nervous system (Supplementary Fig. S6A–F).

### MTOR inhibitors synergize with AKT inhibitor capivasertib in E2A-PBX1^+^ ALL cells

When targeting the PI3K/AKT/MTOR pathway, combined inhibition at different levels of the signaling axis often leads to more effective results than single inhibition of just one target [17]. Therefore, we tested if the combination of the most effective drugs targeting different PI3K/AKT/MTOR members shows a synergistic effect on cell proliferation. We combined the ATP-competitive MTOR-inhibitor torin-1 with the AKT inhibitor capivasertib. Cells were treated with the two drugs simultaneously for four days, then CellTiter-Glo viability assays were performed.

**Fig. 4:**
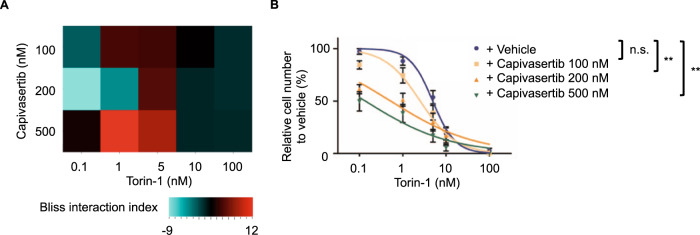
Synergistic effect of the MTOR inhibitor torin-1 and the AKT inhibitor capivasertib. **A** Heatmap representation of Bliss interaction index for RCH-ACV cells treated with the MTOR inhibitor torin-1 and the AKT inhibitor capivasertib. Data represent the mean of three independent experiments. **B** Titration curve for RCH-ACV cells treated with torin-1 in combination with vehicle or capivasertib. Data represent mean ± SEM of three independent experiments. *P* values were calculated using non-linear regression analysis and curves were compared with the sum-of-squares F test. ns not significant, **p < 0.01.

A synergistic effect on cell growth inhibition between capivasertib and torin-1 was identified using the bliss interaction index [18] in RCH-ACV cells (Fig. 4A). The IC_50_ of the drug combination was reduced compared to the single treatment. The synergistic effect of torin-1 and capivasertib was seen in RCH-ACV (Fig. 4B), REH and SEM, but to a lesser extend in 697 cells (Supplementary Fig. S7). An interaction of torin-1 and capivasertib was observed in mouse E2A-PBX1^+^ leukemia cells (Supplementary Fig. S4C). These data suggest that the combination targeted therapy of MTOR and AKT might be more effective than administration of the single drug in a subset of leukemias.

### Modulation of PI3K/AKT/MTOR signaling by MTOR and AKT inhibition in B-ALL

**Fig. 5:**
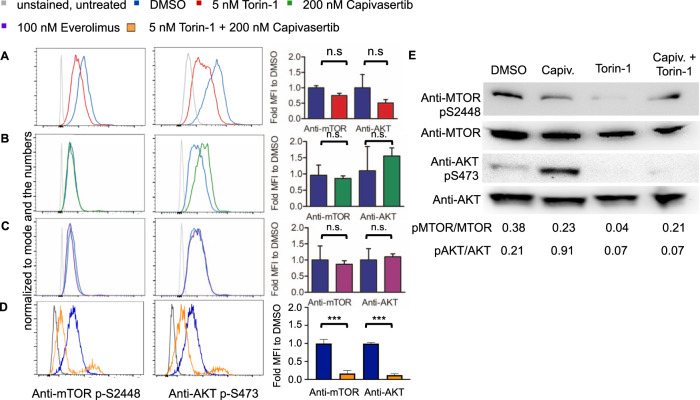
Effects of small molecule inhibitors on the phosphorylation status of key PI3K/AKT/MTOR pathway proteins. Effects of (**A**) torin-1, (**B**) capivasertib (**C**) everolimus and (**D**) combination of torin-1 with capivasertib on p-MTOR Ser2448 and p-AKT Ser473 phosphorylation status in RCH-ACV cells after 15 min treatment. One representative graph is shown. Fold median fluorescence intensity (MFI) change of cells treated with inhibitor compared to vehicle. Data represent mean + SEM of three independent experiments. Statistical analysis was performed by Mann–Whitney *U* test. **E** Western blots using anti-p-MTOR Ser 2448 and anti-p-AKT Ser473 antibodies after the treatment with DMSO (vehicle), Capiv. (capivasertib) 200 nM, torin-1 5 nM and the combination capivasertib 200 nM and torin-1 5 nM. Anti-AKT and anti-MTOR antibodies were used as loading controls. A representative from three experiments is shown. Densitometry values were calculated using ImageJ software.

We next examined the phosphorylation status of p-MTOR (Ser2448) and p-AKT (Ser473) in RCH-ACV cells treated with torin-1, everolimus or capivasertib by phospho-specific flow cytometry. After treatment with torin-1, p-MTOR and p-AKT were reduced compared to vehicle treatment in RCH-ACV cells, whereas cells treated with capivasertib showed increased p-AKT phosphorylation but almost no change of p-MTOR status. As expected, both p-MTOR and p-AKT showed no significant differences after everolimus treatment compared to vehicle in RCH-ACV cells (Fig. 5A–C). The combination of torin-1 and capivasertib decreases significantly the p-MTOR and p-AKT in RCH-ACV cells as shown in phosphoflow (Fig. 5D) and confirmed in immunoblotting assays (Fig. 5E). The phosphorylation of AKT and MTOR was markedly reduced after the in vivo treatment with torin-1 in mouse E2A-PBX1^+^ leukemia cells (Supplementary Fig. S8A, B).

### Metabolic changes after shRNA-mediated knockdown or pharmacological inhibition of MTOR

**Fig. 6:**
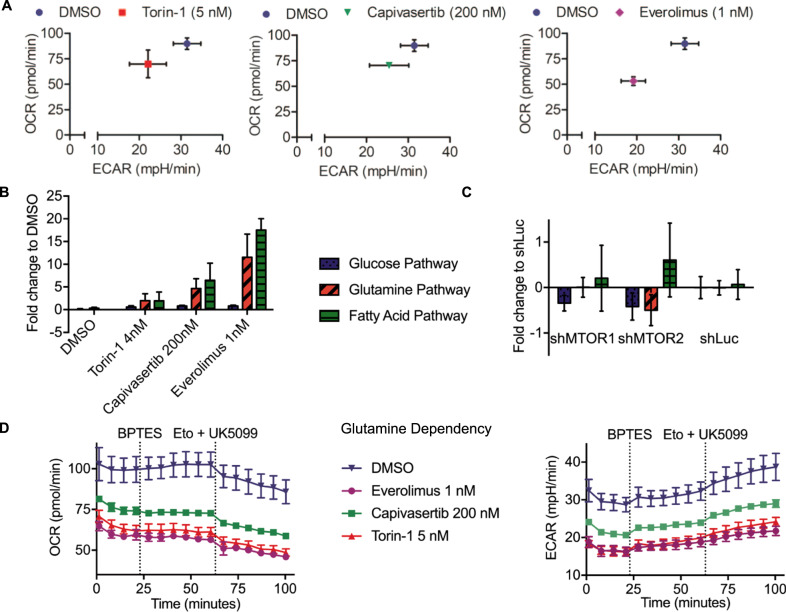
Effects of MTOR and AKT pharmacological inhibition and shRNA-mediated knockdown of MTOR on cell metabolism. RCH-ACV bioenergetics studies were performed in supplemented medium. OCR kinetic reads were taken in 6 min intervals with the first injection after four reads for basal OCR, followed by six reads per inhibitor treatment. **A** Basal OCR and ECAR of RCH-ACV cells after 24 h pretreatment with torin-1, capivasertib or everolimus compared to cells treated with the vehicle. **B** Fold change in dependency on glucose, fatty acids and glutamine for mitochondrial respiration of cells pretreated for 24 h with the indicated inhibitor to DMSO treated cells. Statistical analysis by Student t test assuming a normal distribution. **C** Fold change of shMTOR knockdown cells to shLuc control cells in dependency on glucose, fatty acids and glutamine for mitochondrial fuel oxidation. Dependency was calculated from the difference in basal OCR and OCR after blocking the individual pathway divided by basal OCR less OCR after all inhibitors with sequential injections of 2 mM UK5099, 4 mM etomoxir and 3 mM BPTES, respectively. Data represent mean ± SEM of three independent experiments. **D** OCR (left panel) and ECAR (right panel) plots during the Mito Fuel Flex Test to determine glutamine dependency of RCH-ACV cells pretreated with the indicated substances for 24 h. Dashed lines indicate injection timepoints of the labeled inhibitors. Mean ± SD of one representative experiment with four technical replicates is shown. OCR Oxygen Consumption Rate, ECAR Extracellular Acidification Rate, Eto Etomoxir.

Since MTOR is a major regulator of cell metabolism, we were interested in the metabolic changes after genetic knockdown or pharmacological inhibition of MTOR and upstream pathways in B-ALL cells. Pharmacological inhibition of MTOR and AKT reduced basal oxygen consumption rate (OCR) and extracellular acidification rate (ECAR) of RCH-ACV cells compared to vehicle-treated cells (Fig. 6A; Supplementary Fig. S9).

The Mito Fuel Flex Test determines cellular dependence on glucose, glutamine, and fatty acids to fuel mitochondrial respiration by measuring oxygen consumption rate, when the other substrates are blocked. Pharmacological inhibition of MTOR by torin-1 and everolimus, as well as AKT inhibition by capivasertib, increased the dependence on glucose compared to vehicle-treated cells. The dependency on fatty acid metabolism was increased after both treatments with MTOR inhibitor torin-1 or everolimus and AKT inhibitor capivasertib (Fig. 6B). In contrast, after genetic depletion of MTOR by shRNAs, minimal changes were observed in transduced RCH-ACV cells. They were less dependent on glucose but, consistent with pharmacological inhibition of MTOR, they were more dependent on fatty acids for energy production (Fig. 6C). An increase in glutamine dependence was observed with the treatment of torin-1, capivasertib and everolimus (Fig. 6D).

## Discussion

The PI3K/AKT/MTOR signaling pathway plays a central role in the regulation of metabolism, protein synthesis, proliferation, and apoptosis. Deregulated MTOR signaling is found in a variety of cancer subtypes [19]. In 88% of ALL an hyperactivation of the PI3K/AKT/MTOR signaling pathway is reported and this activation is associated with poor prognosis and chemotherapeutic resistance [20, 21]. Therefore, it represents a promising target for the therapy of leukemia patients.

MTOR is a serine/threonine kinase of the PI3K-like kinase family (PIKK) and functions as a catalytic subunit in the two different multi-protein complexes MTOR complex 1 (MTORC1) and MTOR complex 2 (MTORC2), which are distinguished by their partner proteins, substrates and sensitivity to rapamycin [22]. MTORC1 plays a key role in regulation of nutrient signaling and growth through its downstream targets ribosomal protein S6 kinase 1 (S6K1) and eukaryotic translation initiation factor 4E-binding protein 1 (4E-BP1). RPTOR functions as scaffold protein, which binds the MTORC1 substrates p70S6 kinase and 4E-BP1 at their TOR-signaling (TOS) motif [23–25]. MTORC2 contains RICTOR and mSin1 (also known as MAPKAP1), which are both necessary for the phosphorylation and thus activation of the pro-survival protein kinase B (PKB, also known as AKT1) on Ser 473 residue by MTORC2 [22, 26].

Allosteric MTOR-inhibitors as rapamycin or its derivatives (referred to as rapalogs) like everolimus (RAD001) inhibit MTORC1 by complex formation with FK506-binding protein 12 (FKBP12) and binding to the FKBP12-Rapamycin-Binding (FRB) domain of MTOR, thus blocking the interaction with RPTOR [27].

In this study, we identified and validated MTOR as a key gene on which B-ALL cells are dependent and confirmed the role of MTOR as a regulator of cell proliferation in vitro and in vivo using genetic and pharmacologic techniques. Using shRNA-knockdown cells, we found RICTOR, a part of MTORC2, to be more essential for cell proliferation of RCH-ACV cells than RPTOR. The role of other PI3K/AKT/MTOR signaling pathway members in pre-B-ALL should be analyzed in further studies.

Using a pharmacological approach, we examined the effect of several small molecule inhibitors acting within the PI3K/AKT/MTOR signaling pathway in B-ALL cell lines. Our study confirmed that the ATP-competitive MTOR-inhibitor torin-1 effectively inhibits cell growth in vitro and in vivo in human and mouse ALL cell lines, including E2A-PBX1^+^/preBCR^+^ ALL. It inhibits not only MTORC1, but also MTORC2 activity, as it led to a decreased phosphorylation of its direct substrate S473 p-AKT, which is essential to the full activation of AKT1 [26]. Although torin-1 showed very promising preclinical efficacy in vitro and in vivo in ALL, due to its short in vivo half-life and low oral bioavailability, novel MTOR enzymatic inhibitors with improved pharmacokinetics are urgently needed [28]. A novel MTOR enzymatic inhibitor, called torin-2, has been developed in this direction by Liu and colleagues [29]. Further MTOR inhibitors should be tested for clinical efficacy in future studies.

AZD5363 (capivasertib), an ATP-competitive pan-AKT-inhibitor [30, 31], inhibited cell proliferation particularly in the RCH-ACV and REH cell lines. Surprisingly, increased AKT1 phosphorylation was observed after capivasertib treatment in RCH-ACV cells. This might be explained by the stabilization of the AKT conformation by ATP-competitive AKT-inhibitors, in which both phosphorylated sites are inaccessible to phosphatases [32]. The rapalog everolimus only impairs the MTOR-RPTOR association and thus the interaction of MTOR with its substrates [33]. However, it does not directly inhibit the MTOR kinase activity, thus explaining the lack of phosphorylation changes in S473 p-AKT and S2448 p-MTOR. Since everolimus inhibits cell growth in B-ALL cells, we hypothesize that it is able to inhibit MTORC2 in pre-B cells as suggested by Neri et al. [34].

To improve efficacy and reduce toxicity, it has been tried to combine PI3K/AKT/MTOR inhibitors with cytotoxic chemotherapy in the past. Still, combination with targeted therapies might be more effective in preventing escape via alternative pathways through feedback loops [35]. Therefore, we combined torin-1 with capivasertib and observed a synergistic effect in the inhibition of proliferation of RCH-ACV and REH cells as well as inhibition of phosphorylation of p-AKT and p-MTOR in RCH-ACV cells. The selectivity of the synergism to these two cell lines could be due to their higher sensitivity to capivasertib. Our findings indicate that the combined inhibition of MTOR und AKT could be used as effective targeted therapy in a subset of ALLs.

So far, ALL has been genetically defined very precisely, whereas little is known about its bioenergetic properties. Because MTOR and its complexes are highly involved in the regulation of cell metabolism, we studied the effects of shRNA-mediated knockdown and pharmacological inhibition of MTOR on mitochondrial fuel oxidation. Our findings imply that the pharmacological inhibition or genetic depletion of MTOR causes a metabolic switch from glycolysis to β-oxidation. Notably, changes of genetic depletion of MTOR were less pronounced as compared to the pharmacological inhibition with torin-1. These data are consistent with similar findings in AML cells, which also showed a strong dependence on β-oxidation and in which inhibition of β-oxidation by etomoxir or ST1326, inhibitors of carnitine palmitoyltransferase 1a (CPT1A), resulted in an antiproliferative effect, induction of apoptosis and sensitized the cells to small molecule inhibitors [36–38]. Therefore, future studies should investigate the combination treatment of MTOR targeted therapies and inhibition of fatty acid pathways in B-ALL cells. Exploiting this dependency pharmacologically may help to improve the efficacy of PI3K/AKT/MTOR inhibition in combination therapies.

In summary, we identified MTOR as a key gene, on which pre-B-ALL are dependent on for proliferation and survival in vitro and in vivo and we provided a rationale for the combination of AKT and MTOR inhibitors in the treatment of a subset of pre B-ALL. Furthermore, we showed phosphorylation and metabolic changes in pre-B-ALL after inhibition and depletion of PI3K/AKT/MTOR pathway.

## Materials and methods

### Materials

#### shRNA library screen for cell proliferation in B-ALL

The shRNA screen was performed as described elsewhere [16, 39, 40]. Three shRNA lentivirus sublibraries containing a total of about 106,249 shRNA sequences and targeting 3,158 human genes (about 25 shRNAs for most genes and 50 shRNAs for kinases/phosphatases) and containing 13,774 negative control shRNAs were used to transduce RCH-ACV cells in four pools. After puromycin selection and expansion, RCH-ACV cells stably expressing pooled shRNAs were cultured for 12 days. Cells were split every three days. The influence of each individual shRNA on cell proliferation was calculated using Mann–Whitney *U* test (Supplementary Table S1) and casTLE, a novel statistical framework for cas9 and shRNA screening technologies (Supplementary Table S2, ref. [41], v1.0 available at https://bitbucket.org/dmorgens/castle). The enrichment score for each shRNA was normalized by the distribution of enrichment scores of the negative control shRNAs [39]. Candidate shRNAs shown to confer sensitivity or resistance are shown in Supplementary Table S1 and S2. Bioinformatics analysis of genes included in the KEGG pathway MTOR (hsa04150) and included in the shRNA screen is shown in Supplementary Table S3.

#### shRNA downregulation, lentiviral transfection, and competition growth assays

Individual shRNA sequences (Supplementary Table 4) were cloned into the BstXI site of the p309 lentiviral vector [39], for stable transduction in human ALL cell lines. Lentivirus were generated by co-transfection of shRNA constructs with pCMV-dR8.2 (packaging) and pCMV-VSVG (envelope) plasmids into actively growing HEK293T cells using TurboFect (Invitrogen). Human leukemia cells were transduced with viral supernatant, collected 48 h after transfection and supplemented with 4 µg/ml polybrene, by spinoculation (2500 rpm, 37 °C for 3 h) and cultured for two days prior to puromycin-selection. After four days of selection with 1 µg/ml puromycin (Invitrogen), cells with shRNA knockdown were mixed 1:1 with cells containing a control vector and cultured for 30 days. Co-cultured mCherry^+^ and GFP^+^ transduced cells were monitored over time by flow cytometry. To confirm the knockdowns of the single shRNAs, we performed immunoblotting and quantitative RT-PCR after puromycin selection.

### Cell lines

E2A-PBX1^+^ B-ALL cell lines RCH-ACV and 697, as well as E2A-PBX1^-^ B-ALL cell lines REH and SEM were obtained from DSMZ (Braunschweig, Germany) in 2013, and were authenticated in 2020 in DSMZ. They were monitored to exclude mycoplasma contamination regularly. Culturing of human cells was performed as previously described [7]. RPMI-1640, IMDM, DMEM, and Opti-MEM mediums were obtained from Thermo Fisher (Waltham, MA, USA). Torin-1, everolimus (RAD001), ipatasertib (GDC-0068), alpelisib (BYL719), capivasertib (AZD5363) and idelalisib (CAL-101) were from Selleck Chemicals (Houston, TX, USA). FBS was from Sigma-Aldrich (St. Louis, WIS, USA).

### Treatment with small molecule inhibitors

For cell line growth curve experiments, 25 × 10^4^ B-ALL cells/ml were seeded in 24-well plates and treated with the indicated small molecule inhibitors in increasing concentrations immediately after seeding. The vehicle control wells were treated with a volume of dimethyl sulfoxide (DMSO) equal to the volume of drug solution added to the other treatments. After 4 days of treatment, the number of viable cells was counted with trypan blue exclusion assay (Thermo Fisher) or determined by ATP quantitation with CellTiter-Glo Luminescent Cell Viability Assay (Promega). Combination indices were calculated by CIM Miner (National Cancer Institute NCI).

### Colony-forming assays and in vitro drug treatment of murine E2A-PBX1^+^/preBCR^+^ leukemia cells

Mouse leukemia cells (25,000/well) were cultured in methylcellulose medium (M3234, StemCell Technologies) supplemented with 10 ng/mL IL7 (Miltenyi Biotec). Colonies were counted after seven days. Leukemia cells were cultured in the presence of vehicle (DMSO) or torin-1, everolimus or capivasertib (Selleck Chemicals, Houston, TX, USA) at the concentrations indicated.

### Bone marrow transplantation assays and in vivo drug treatment

Secondary bone marrow transplantation assay using 1000 murine E2A-PBX1 + /preBCR+ leukemia cells per recipient after sublethal irradiation (6 Gy) was described elsewhere [6, 7]. For in vivo treatment, a cohort of five C57BL/6 female mice with an age between 10–14 weeks were treated daily, intraperitoneally, starting the 7 days after transplantation with vehicle (30% PEG300, 5%Tween 80, 5% DMSO dissolved in PBS) or torin-1 (20 mg/kg b.w./day, Selleck Chemicals, Houston, TX, USA) as described previously [28]. Disease-free survival was defined as the time from transplantation to the time when the mice showed signs of illness, including general lymphadenopathy, lethargy, weight loss, and shivering. Moribund mice were euthanized. No animals were excluded from the analysis. No randomization method was used to allocate the animals to an experimental group. The investigators were not blinded to the group location during the experiment. All experiments were performed in accordance with relevant guidelines and regulations and were approved by the “Regierungspräsidium Freiburg“ (Nr. G-17/148).

### Western blot analysis

Western blots were performed as previously described [6]. Proteins transferred to nitrocellulose membranes (Amersham) were immunodetected with mouse anti-Vinculin Antibody (7F9) (sc-73614, Santa Cruz Biotechnology), rabbit anti-mTOR antibody (ab2732, Abcam), mouse anti-Raptor antibody (10E10) (sc-81537, Santa Cruz Biotechnology), rabbit anti-Rictor antibody (53A2) (2114, Cell Signaling Technology). Antibodies used for phosphorylation analysis: rabbit anti-mTOR Ser2448 (#2971, Cell Signaling Technology), rabbit anti-AKT Ser473 (#9271 T, Cell Signaling Technology), rabbit anti-mTOR (#2972 S, Cell Signaling Technology), rabbit anti-AKT (#9272 S, Cell Signaling Technology). Secondary antibodies horse anti-mouse IgG (7076, Cell Signaling Technology) and goat anti-rabbit IgG (7074, Cell Signaling Technology) coupled to HRP were used.

### Phospho-flow analysis and flow cytometry

Phospho-flow analysis and flow cytometry analysis were performed as described previously [6]. FACS analysis was performed using a LSR Fortessa Cell Analyzer (BD Biosciences), with 10,000 events being acquired for each sample. Alexa 647 p-MTOR (Ser2448) (clone O21-404) and PE p-AKT (Ser473) (clone M89-61) for flow cytometry analysis were purchased from BD Biosciences (Franklin Lakes, NJ, USA).

### qRT-PCR

RNA was isolated using the NucleoSpin RNA kit (Macherey-Nagel) and cDNA synthesized using SuperScript III Reverse Transcriptase (Thermo Fisher) following the manufacturer’s instructions. Relative expression was quantified using an LightCycler 480 II Thermocycler (Roche) with TaqMan gene expression assays from Thermo Fisher: ACTB (Hs01060665_g1), MTOR (Hs00234508_m1), RICTOR (Hs00380903_m1), RPTOR (Hs00375332_m1) and Light Cycler 480 Probes Master (Roche) at an annealing temperature of 60 °C. All signals were quantified using the ΔC_t_ method and normalized to the ΔC_t_ values of *ACTB* gene expression levels. All PCR assays were performed three times and the results shown were from samples analyzed in triplicate in each experiment.

### Metabolic fuel flex assays

For measurements of oxygen consumption rate (OCR) and extracellular acidification rate (ECAR) the Seahorse XF Extracellular Flux Analyzer Fe96 (Agilent, Santa Clara, CA, USA) was used. It measures the concentration of free protons and oxygen above a cell-monolayer in real-time. Cells were seeded at 120,000/well into a Poly-D-lysine-coated XF 96-well microplate and centrifuged to adhere to the bottom of the well. XF RPMI Medium was supplemented with 2 mM L-glutamine, 10 mM D-glucose and 1 mM sodium pyruvate. Mito Fuel Flex Assays were performed following the manufacture’s protocol. Briefly, this assay determines cellular dependence on the three major metabolites fueling mitochondrial metabolism (glutamine, fatty acids, and glucose) by inhibiting the individual substrate import or capacity to metabolize it, when the others are blocked. 2 µM UK5099 were used to inhibit the mitochondrial pyruvate carrier, 3 µM BPTES to inhibit glutaminase and 4 µM Etomoxir for carnitine palmitoyltransferase 1 A inhibition.

Baseline OCR and ECAR were measured over 24 min, then treatment 1 was injected. After 36 min of measurements, treatment 2 was injected and OCR and ECAR were measured again over 36 min. Data were analyzed using Seahorse XF Mito Fuel Flex Test Report Generator.

Calculations and composition of inhibitor treatments are shown in Table 1. A representative course of the Mito Fuel Flex Assay is shown in Fig. 6D and Supplementary Fig. S9.

**Table 1:** Metabolic tests and their associated inhibitors.

Dependency test	Treatment 1	Treatment 2
Glutamine dependency	BPTES	Etomoxir + UK5099
Fatty acid dependency	Etomoxir	BPTES + UK5099
Glucose dependency	UK5099	BPTES + Etomoxir

Calculation of dependency

$$Dependency\left( {{{\mathrm{\% }}}} \right) = \left[ {\frac{{baseline\;OCR - target\;inhibitor\;OCR}}{{baseline\;OCR - all\;inhibitors\;OCR}}} \right] \times 100$$

### Statistical analysis

All experiments were performed in triplicate. Data are presented as mean ± SD or SEM of three experiments. Statistical differences between two groups were determined by unpaired two-sided Mann–Whitney *U* test or by Student *t* test. A *p* < 0.05 was considered statistically significant. Survival curves were analyzed by log-rank (Mantel–Cox) test. Titration and cell growth curves were analyzed using non-linear regression analysis and compared with the sum-of-squares *F* test. Statistical analysis and graphs were performed using GraphPad Prism software, version 9 (GraphPad Inc.).

## Supplementary information

Supplementary Figures

Supplementary Tables

## Data Availability

The data generated during and/or analyzed during the current study are available upon reasonable request from the corresponding author.
