# Supplementary material for: Functional characterization of the PI3K/AKT/MTOR signaling pathway for targeted therapy in B-precursor acute lymphoblastic leukemia
Source: Cancer Gene Ther. 2022 Jul 6;29(11):1751–60. doi: 10.1038/s41417-022-00491-0 (PMC9663301; doi:10.1038/s41417-022-00491-0)
Supplement: Supplementary file 1 — Supplementary Figures [file 41417_2022_491_MOESM1_ESM.pdf]

**Supplementary Material**

**Suppl. Table S1, S2 and S3 (see excel file)**

| | | |
|---|---|---|
| shLuc | FW | AAGCTGTTTCTGAGGAGCCTAGTGAAGCCACAGATGTAGGCTCCTCAGAAACAGCTCTGCC |
| | RW | GAGCTGTTTCTGAGGAGCCTACATCTGTGGCTTCACTAGGCTCCTCAGAAACAGCTTCGCT |
| MTOR shRNA 1 | FW | CCAATGTGCAGGATCTTCCCACTAGTGAAGCCACAGATGTAGTGGGAAGATCCTGCACATTGATGCC |
| | RW | TCAATGTGCAGGATCTTCCCACTACATCTGTGGCTTCACTAGTGGGAAGATCCTGCACATTGGCGCT |
| MTOR shRNA 2 | FW | CTAGCTGTGGAATCTGACGGCTTAGTGAAGCCACAGATGTAAGCCGTCAGATTCCACAGCTAATGCC |
| | RW | TTAGCTGTGGAATCTGACGGCTTACATCTGTGGCTTCACTAAGCCGTCAGATTCCACAGCTAGCGCT |
| RICTOR shRNA 10 | FW | ACCGTATACTCCTTCGCAATAGTGAAGCCACAGATGTATTGCGAAGGAGTATACGGCTGCC |
| | RW | GCCGTATACTCCTTCGCAATACATCTGTGGCTTCACTATTGCGAAGGAGTATACGGTCGCT |
| RPTOR shRNA 2 | FW | ACTCTTGCTCAGATGCCTTTAGTGAAGCCACAGATGTAAAGGCATCTGAGCAAGAGGTGCC |
| | RW | CCTCTTGCTCAGATGCCTTTACATCTGTGGCTTCACTAAAGGCATCTGAGCAAGAGTCGCT |
| RPTOR shRNA 4 | FW | ACAGGTGCTGTTAAGCCAATAGTGAAGCCACAGATGTATTGGCTTAACAGCACCTGCTGCC |
| | RW | GCAGGTGCTGTTAAGCCAATACATCTGTGGCTTCACTATTGGCTTAACAGCACCTGTCGCT |

**Suppl. Table 4. Sequences of the oligonucleotides**

FW forwards, RW reverse

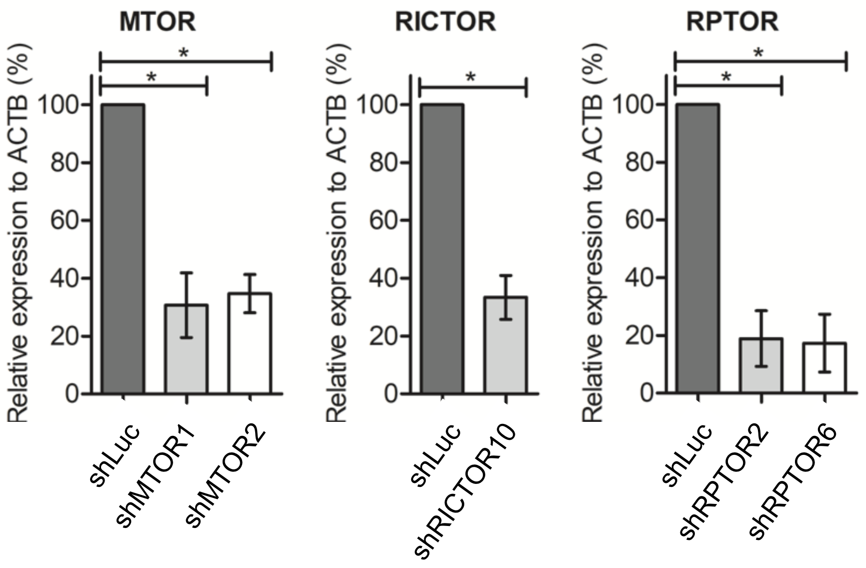


**Supplementary Figure S1. Graph shows relative expression to *ACTB* of *MTOR*, *RICTOR* and *RPTOR* by qRT-PCR after shRNA-mediated knockdown.** Data represent the mean ± SEM of three independent experiments. Statistical analysis by Student t test (*, P < 0.05).

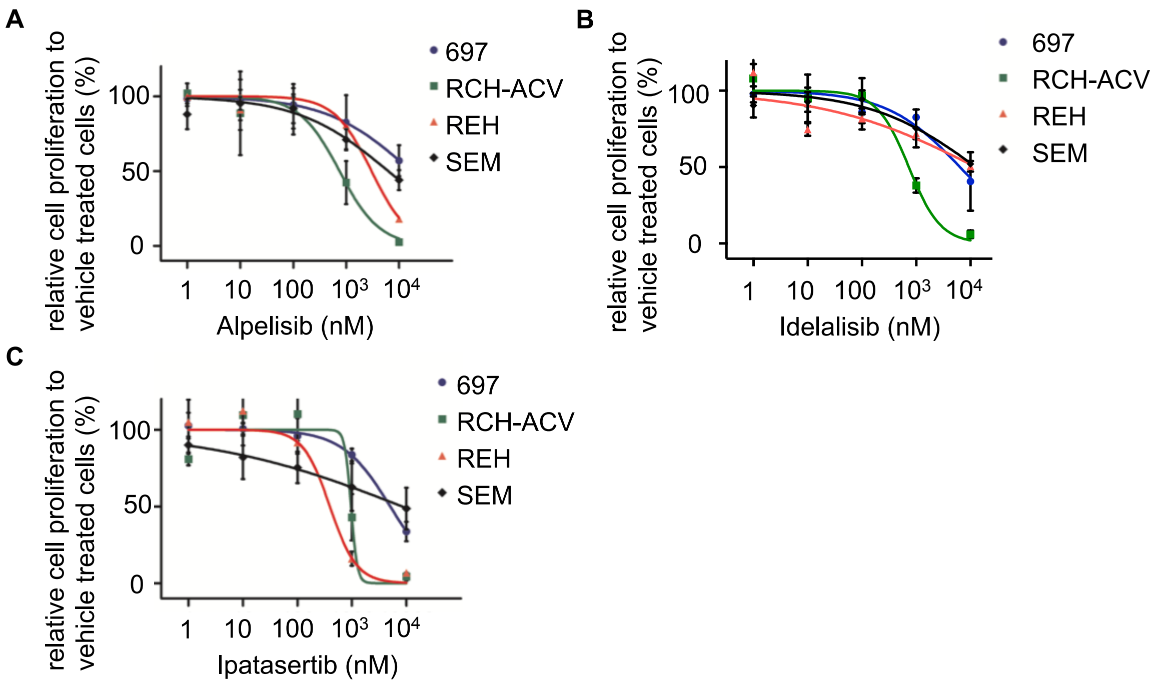


**Supplementary Figure S2. Titration curves for human B-ALL cells** cultured with increasing concentrations of the specific PI3K inhibitors **(A)** alpelisib and **(B)** idelalisib and the AKT inhibitor **(C)** ipatasertib. Viable cells were counted after 4 days by trypan blue exclusion assay. Data are represented as mean ± SEM of three independent experiments.

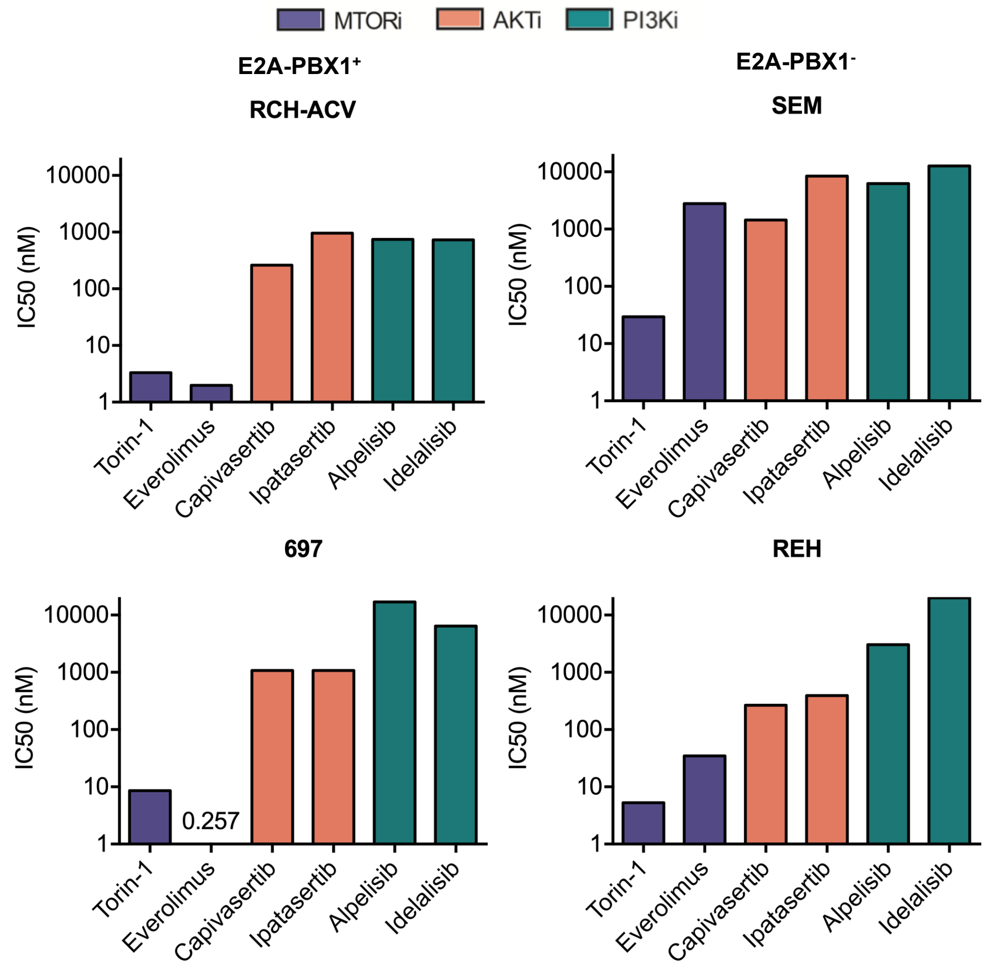


**Supplementary Figure S3. Half inhibitory growth concentrations (IC50) of different small molecule inhibitors targeting the PI3K/AKT/MTOR signaling axis tested in E2A-PBX1$^{+}$ and E2A-PBX1$^{-}$ cell lines.** Cells were treated for 4 days with the specific inhibitors. Data represent IC50 calculated by GraphPad Prism software after three independent experiments. MTORi: MTOR inhibitor; AKTi: AKT inhibitor; PI3Ki: PI3K inhibitor

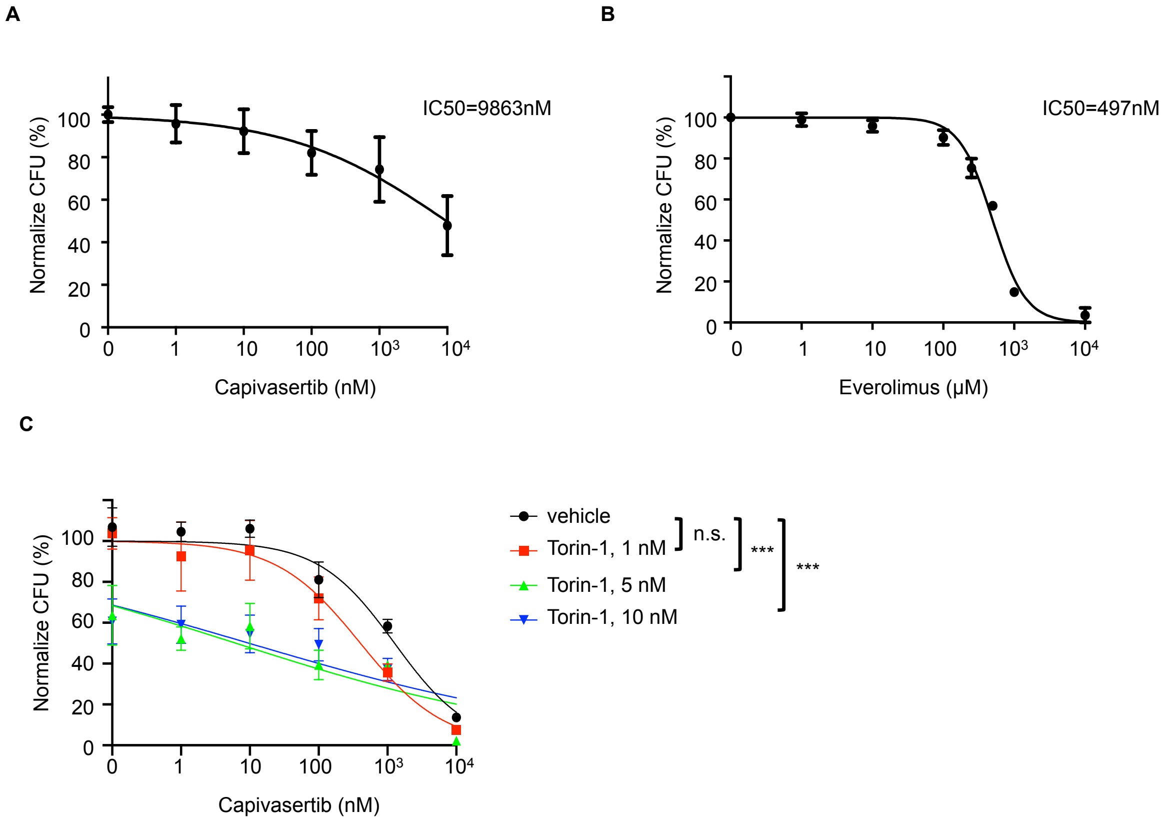


**Supplementary Figure S4. Titration curves for mouse E2A/PBX1$^+$ leukemia cells** cultured with increasing concentrations of **(A)** capivasertib, **(B)** everolimus and **(C)** the combination of torin-1 and capivasertib. Leukemia cells were cultured in methylcellulose and colonies were counted after 3-5 days. Data are represented as mean ± SEM of three independent experiments. *P* values were calculated using non-linear regression analysis and curves were compared with the sum-of-squares F test. ns: not significant, *** p<0.001. CFU, colony forming units.

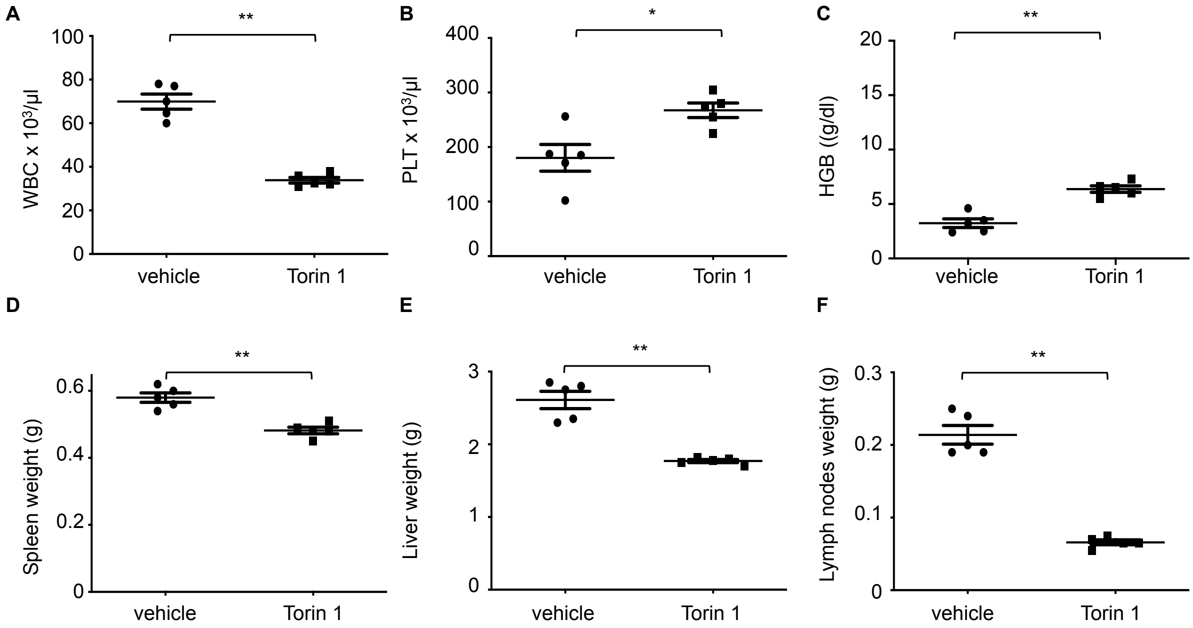


**Supplementary Figure S5. Features of mice transplanted with murine E2A-PBX1$^{+}$/PreBCR$^{+}$ leukemias and treated with torin-1.** Recipient mice were transplanted with 1.000 murine E2A-PBX1$^{+}$/PreBCR$^{+}$ leukemia cells after sublethal irradiation. *In vivo* treatment was performed with vehicle or torin-1(20 mg/kg b.w./d) starting at day 8 after transplantation. Each cohort contains 5 mice. Following characteristics were compared: **(A)** white blood cells, WBC; **(B)** platelets, PLT; **(C)** hemoglobin, HGB; **(D)** spleen weight; **(E)** liver weight and **(F)** lymph nodes weight. Statistical analysis was performed by two-sided Mann-Whitney U test. Scatter dot plots represent mean ± SEM. * $p<0.05$; **, $p<0.01$

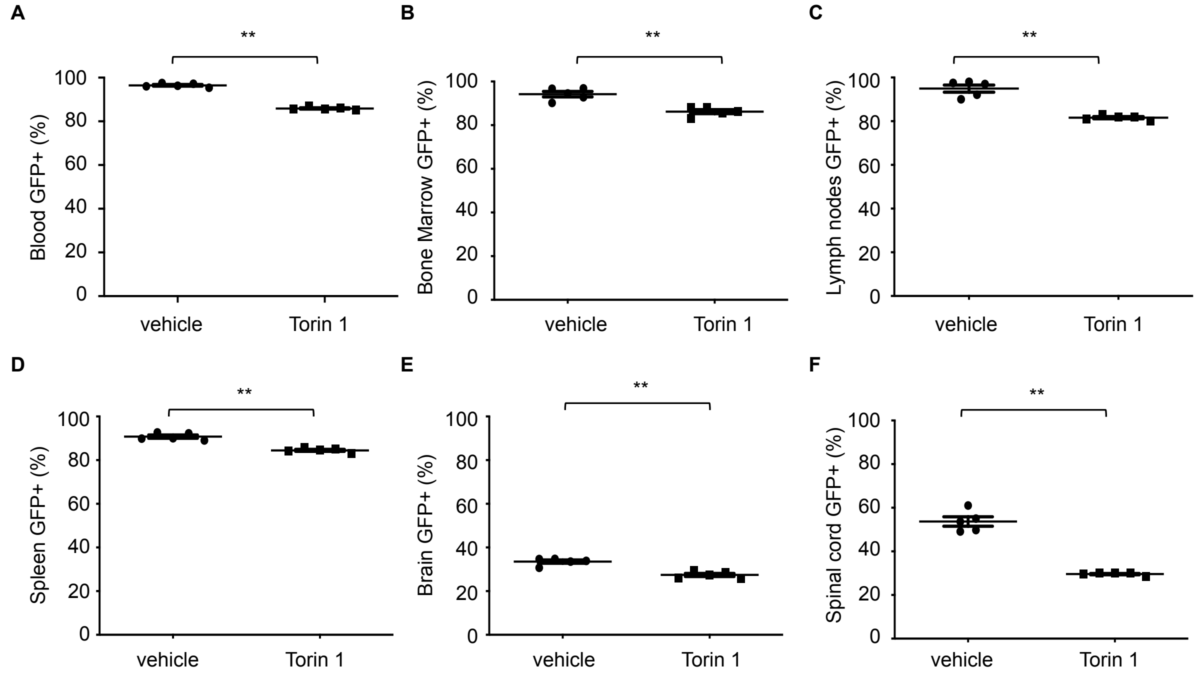


**Supplementary Figure S6. Tissue Infiltration of murine E2A-PBX1$^+$/PreBCR$^+$ leukemia cells after transplantation in recipient mice and *in vivo* treatment with torin-1.** GFP+ murine E2A-PBX1$^+$/PreBCR$^+$ leukemia cells were assessed in following tissues and organs by flow cytometry: **(A)** blood; **(B)** bone marrow; **(C)** lymph nodes; **(D)** spleen; **(E)** brain and **(F)** spinal cord. Each cohort contains 5 mice. Statistical analysis was performed by two-sided Mann-Whitney U test. Scatter dot plots represent mean ± SEM. **, $p<0.01$

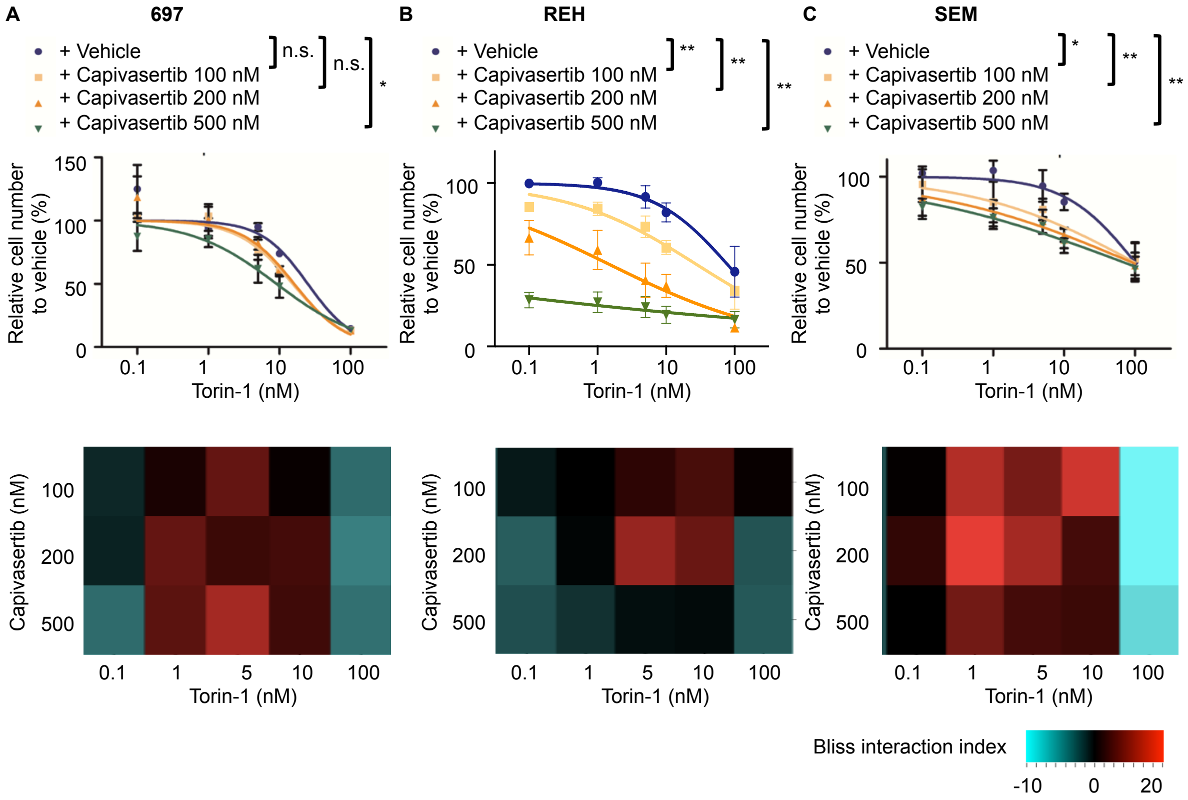


**Supplementary Figure S7. Combinational treatment with capivasertib and torin-1**. *Upper panels,* titration curves are shown for **(A)** 697, **(B)** REH and **(C)** SEM cells. Statistical analyses were performed using non-linear regression analysis and curves were compared with the sum-of-squares F test. ns: not significant, *, p<0.05, ** p<0.01, *** p<0.001. Data represent mean ± SEM of three independent experiments. *Lower panels*, Heatmap representation of Bliss interaction index for 697, REH and SEM cells treated with the MTOR inhibitor torin-1 and the AKT inhibitor capivasertib. Data represent the mean of three independent experiments.

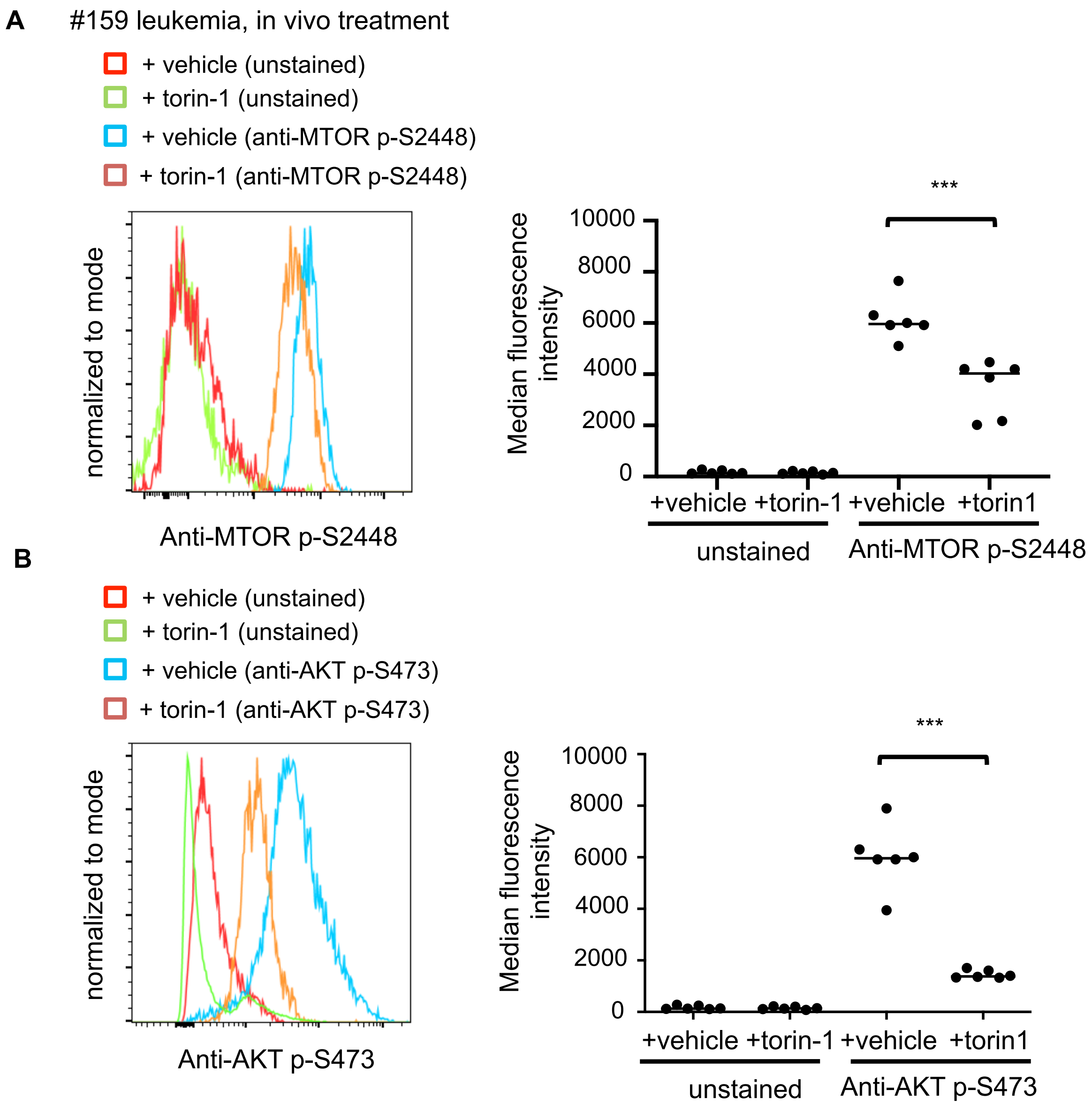


**Supplementary Figure S8. Effects of *in vivo* treatment with torin-1 on the phosphorylation status of MTOR and AKT on leukemia cells from bone marrow.** Effects of torin-1 on **(A)** Ser2448 p-MTOR and **(B)** Ser473 p-AKT phosphorylation status were assessed by phosphoflow in mouse E2A-PBX1$^+$ leukemia cells infiltrating bone marrow after *in vivo* treatment with vehicle or torin 1 (20 mg/kg b.w. per day). GFP+ cells expressing E2A-PBX1$^+$ were gated for the analysis. One representative histogram is shown. Change of median fluorescence intensity (MFI) of cells treated with inhibitor was compared to vehicle as well as unstained cells as controls. Each dot represents a measurement of three independent experiments from two treated mice per cohort. Statistical analysis was performed by two-sided Mann-Whitney U Test. *** p<0.001.

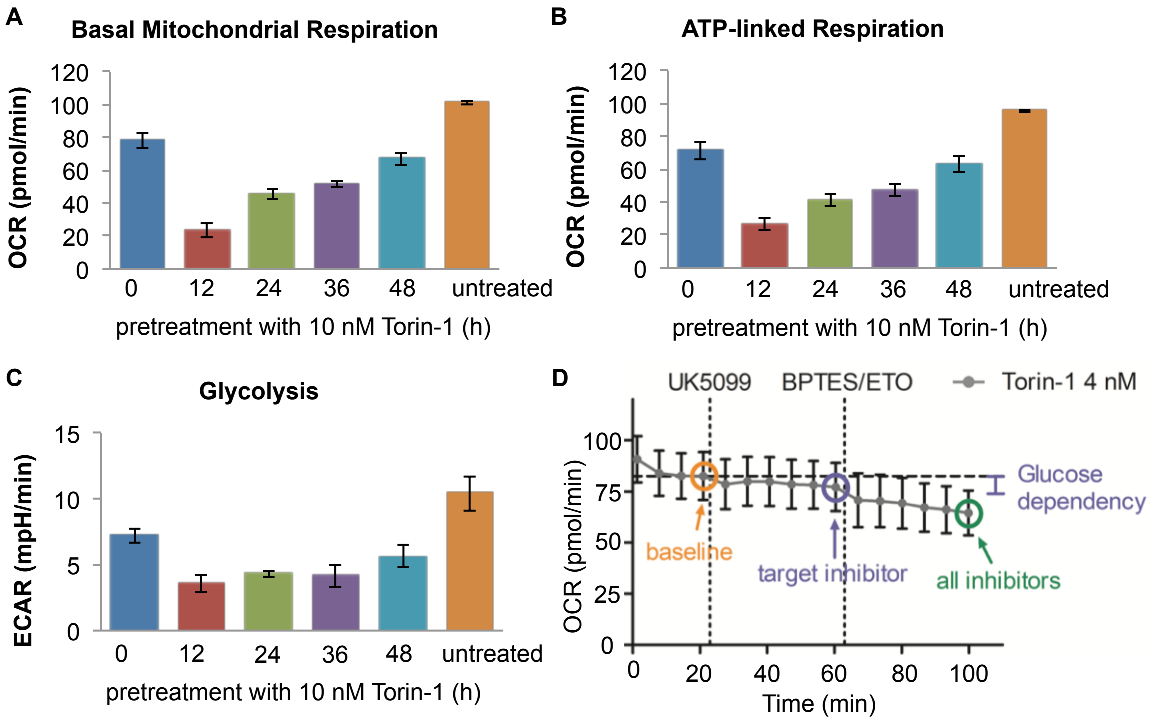


**Supplementary Figure S9. Oxygen metabolism in RCH-ACV cells treated with torin-1. (A-C)** Time course of mitochondrial respiration rates of RCH-ACV cells pretreated with 10 nM torin-1. Samples “0” were treated immediately before starting metabolic measurements in the Seahorse XFe96 Analyzer. Data represent mean ± SD of at least three replicates in one experiment. **(A)** Basal mitochondrial respiration. **(B)** ATP-linked respiration calculated after serial injections of 2 µM oligomycin, 1.25 µM FCCP and 1 µM Rotenone/antimycin A. **(C)** Glycolysis rate calculated after serial injection of 10 mM glucose, 1 µM oligomycin and 50 mM 2-deoxy-glucose. Cells were seeded in glucose-free medium for this assay. **(D)** Representative Mito Fuel Flex Assay with RCH-ACV cells following torin-1 pretreatment to determine glucose dependency. Dashed lines show injection times of the labeled inhibitors. The circled measuring points were used to calculate the dependency. Data represent mean ± SD. OCR: Oxygen Consumption Rate; ECAR: Extracellular Acidification Rate; ETO: etomoxir
